# Dentin Hypersensitivity Treated With Diode Laser and Aminefluoride: A Randomized Clinical Trial

**DOI:** 10.1155/ijod/1399815

**Published:** 2025-11-08

**Authors:** Domenico Aiello, Mario Romeo, Melania Reda, Paolo Zampetti, Sergio Paduano, Andrea Scribante

**Affiliations:** ^1^Department of Health, University “Magna Graecia” of Catanzaro, Viale Europa, Loc. Germaneto, Catanzaro 88100, Italy; ^2^Consultant, Via Lazio 8, Policoro 75025, Italy; ^3^Consultant, Cosenza 87100, Italy; ^4^Section of Dentistry, Department of Clinical, Surgical, Diagnostic and Pediatric Sciences, University of Pavia, Piazzale Golgi 2, Pavia 27100, Italy

**Keywords:** dentin hypersensitivity, diode laser, fluoride, hypersensitivity treatment, laser treatment

## Abstract

**Objectives:**

The aim of this trial was to evaluate the effect of amine fluoride (AF) gel with or without diode laser application on pain due to dentin hypersensitivity (DH).

**Methods:**

The numerical rating scale (NRS) index was used to assess the pain experienced by each patient. The pain response was elicited by a thermal stimulus (cold air blast). The sample included 43 patients, for a total of 86 dental elements with a split-mouth design, 27 females and 16 males, with an age range between 18 and 68 years. The teeth were divided into two groups. The treatment group, consisting of 43 teeth, was treated with 980 nm diode laser (doctor smile wiser by Lambda s.p.a. Via dell'Impresa, 1,36040 Brendola VI, Italy) and AF gel (Elmex Dental Gel, Colgate-Palmolive company, New York, 300 Park Ave, United States); the control group, consisting of 43 teeth, was treated with AF gel only. Only one treatment session was performed for each group. Follow-up visits were scheduled for each patient at 1 week, 1, 3, and 6 months. At these follow-up sessions, the pain response was reassessed using the NRS index.

**Results:**

The results of this study showed a significant reduction in pain in both groups (*p* < 0.05). However, teeth treated with laser therapy showed significantly better pain control over time compared with conventional treatment (*p* > 0.05). AF therapy combined with laser therapy was shown to be more effective in reducing pain than AF alone, both immediately after therapy and in the long term (*p* < 0.05).

**Conclusions:**

The laser combined with the AF gel guarantees a better result on reduction of tooth sensitivity compared to the AF gel alone.

## 1. Introduction

Dentin hypersensitivity (DH) is a clinical situation characterized by pain in the coronal dentin area in response to thermal, tactile, osmotic, or chemical stimuli, and this phenomenon can not occur without dentin exposure [1]. The average prevalence in the world population is between 4% and 57% of the population, with a high prevalence in subjects between 20 and 40 years of age, without significant differences between men and women [2]. The etiopathogenesis of this condition is multifactorial, and predisposing factors include physical abrasions, acid erosions, trauma, and iatrogenic factors [3]. The prevalence of DH increases significantly when it is associated with periodontal disease, with a percentage of 60%–98% [4–6]. Patients with DH report brief acute pain localized to one or more dental elements [7]. The teeth most affected are the upper bicuspid teeth, followed by the upper first molars and the lower incisors, with the vestibular–cervical zone appearing to be the most vulnerable [8].

Several theories have been proposed to explain the pain sensation of DH, but the most accepted is the hydraulic conductivity of dentin, also known as the “hydrodynamic theory.” According to this theory, pain results from the movement of fluid within dentin tubules, stimulating structures of the dentin/pulp complex in response to changes in pulpal pressure at the level of the odontoblast layer [9].

Various products have been used throughout history to treat DH pain, both at home and in the dental profession [10]: fluorinated substances, glutaraldehyde, potassium salts, strontium salts, arginine/calcium, amorphous calcium phosphate, but also some natural substances, such as propolis, have been used with mixed success and always for a limited period of time [10–14].

Some studies have shown satisfactory results with laser radiotherapy in dentistry [15, 16]. This treatment provides immediate results due to its analgesic effect and long-lasting effects due to its ability to stimulate the formation of secondary dentin [17]. The exact mechanism of action of lasers in DH is not clearly understood, although several theories have been proposed. For low power lasers (e.g., GaAlAs), irradiation may have a photobio-modulating effect on cellular activity, increasing the deposition of tertiary dentin by odontoblastic cells. Medium power lasers (e.g., Er:YAG, Nd:YAG, and Er, Cr:YSSG) may reduce or obliterate the dentinal tubules. For Er:YAG and Er, Cr:YSSG, the effectiveness in reducing DH is thought to be related to the thermomechanical ablation mechanism and the high absorption of their wavelengths by water [18]. These are physical methods that, although operating in different wavelength bands, cause the dentin-pulp complex to respond to irradiation by obliterating the dentinal tubules through a specific biological mechanism [11]. Diode laser irradiation with a wavelength of 700–980 nm can induce a photobiomodulating effect, increasing the cellular metabolic activity of odontoblasts and obliterating the dentinal tubules with intensification of tertiary dentin production [19]. There are currently few studies that compared the effect of flouride base desensitive agent alone and in combination with laser [20, 21]. The aim of this randomized controlled clinical trial was to evaluate the effect of amine fluoride (AF) gel alone or in combination with 980 nm diode laser in the treatment of pain due to DH and the change in pain sensitivity over time. The null hypothesis of the study was that there would be no significant difference in numerical rating scale (NRS) scores over time between the two treatments.

## 2. Materials and Methods

### 2.1. Study Design

This is a single-centre, split-mouth, randomized controlled trial with a 1:1 allocation ratio. “The trial was conducted in accordance with the tenets of the Declaration of Helsinki for human experimentation on date 15/07/2020.” The CONSORT statement was followed for this study. Written informed consent was obtained from the parents/guardians of the subjects involved. The protocol was approved by the Ethics Committee of the University of Catanzaro “Magna Graecia” with identification code 254. Full trial protocol along with sources of potential biases and study generalizability is available and registered on clinicaltrials.gov (Registration Number: NCT06202495 January 1, 2024).

Operator blinding was not possible for the specific clinical nature of the study.

### 2.2. Sample Size

Sample size calculation (alpha = 0.05; power = 95%) was conducted for two independent study groups and a continuous primary endpoint with a split-mouth design. Basing on previous study [22], concerning the variable NRS, an expected mean of 5.29 was hypothesized, with a standard deviation of 1.41. The expected difference between the means was supposed to be 0.99 [22]; therefore, 43 patients were required.

Patients are selected from A.O. Mater Domini, U.O. Odontoiatria, University of Catanzaro “Magna Græcia.”

The sample requires the following exclusion and inclusion criteria.

### 2.3. Inclusion Criteria

- Teeth in good periodontal health, normal probing sulcus depth (from 1 to 3 mm). Teeth showed maximum Miller Class I recessions.
- Good oral hygiene (G.I. = 0).
- DH: NRS ≥ 3.

### 2.4. Exclusion Criteria

- Teeth local disease or fractures in treatment area.
- Irreversible teeth necrosis or pulpitis.
- Definitive restorations or veneers.
- Recent use of painkillers or anti-inflammatory drugs.
- Treatment for DH less than 6 months before the study start.
- Therapies contraindication.

### 2.5. Methods

**Figure 1:**
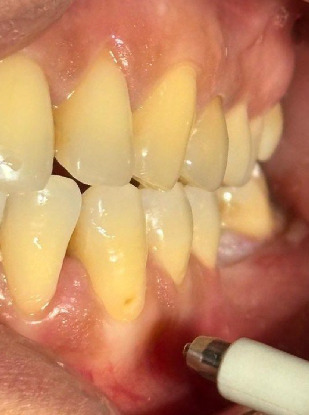
Induction of the thermal stimulus.

The sample consisted of 43 patients, 27 females and 16 males, aged between 18 and 68 years, with a total of 86 teeth analyzed, Table 1 (the two most sensitive teeth were included for each; to facilitate experimentation, only the canine, incisors, and premolars of the two arches were evaluated). The teeth were subjected to a thermal stimulus with an air blast test, 5 s of cold air blown at a distance of 0.2 cm for each dental element (Figure 1), at time T0, before the start of therapy. DH was assessed using the NRS, where 0 means no pain and 10 means unbearable pain; this was chosen over the visual analog scale (VAS) because it allows a more unidimensional assessment of pain [1, 23, 24]; for each patient, the tooth with the highest NRS on the left and right sides was selected. After this assessment, a professional hygiene, education, and motivation session was carried out for each patient, and then the teeth were randomly divided into two groups (*N* = 43) using a split-mouth method.

### 2.6. Trial and Control Group

**Figure 2:**
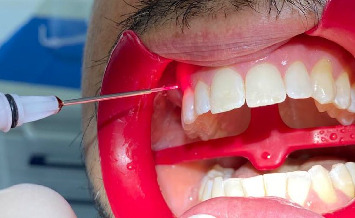
Diode laser application during the trial procedure.

By means of a permuted block randomization table provided by the data analyst, 43 patients were randomized into groups, according to a split-mouth design. An operator enrolled the participants and executed the professional oral procedures. Based on previously prepared sequentially numbered, opaque, sealed envelopes (SNOSE), an assistant assigned patients to the respective treatment. The order was randomized. The data analyst was blinded. The treatment group was treated with AF gel (Elmex Dental Gel, Colgate-Palmolive company, New York, 300 Park Ave, United States). AF was applied according to manufacturer instructions and immidiatly prior to activation of the laser. Laser was a 980 nm diode laser (doctor smile wiser by Lambda s.p.a. via dell'Impresa, 1, 36040 Brendola VI, Italy) 1.5 W, pulsed mode, 2 mm from the tooth surface (Figure 2); for the treatment, 400 μm diameter and 5 mm long tips were used for 4 min as the maximum recommended by the manufacturer. The control group was treated with AF gel only for 4 min according to manufacturer's instuctions. A single treatment session was performed for each group. After the treatment session, pain was assessed using the NRS scale: 1 week (T1), 1 (T2), 3 (T3),and 6 (T4) after treatment.

Enrollment began on January 15, 2024 and ended on July 30, 2024.

No changes to the methods after trial commencement occurred.

A single examiner (LS) selected patients by administering an anamnestique questionnaire and performing the clinical evaluation to ensure that all subjects fulfilled all the inclusion criteria.

Randomized sequence was generated with computer software (R version 3.1.3, R Development Core Team, R Foundation for Statistical Computing, Wien, Austria).

In order to ensure equal distribution, all eligible patients were randomly allocated in either the control or the trial group by means of the randomization table.

For allocation concealment, the operator in charge of the laser and fluoride or fluoride alone groups consulted the randomization list and performed the corresponding procedure. The randomization list was generated and held securely in a remote location.

For the implementation of the randomization, the allocation sequence was generated by the first operator (DA). He was blinded to clinical visits and measures. The discussion with the patients explaining study design was performed by an operator (SP) that was blinded to both clinical measures and randomization list generation. Another clinician (MR) enrolled participants and assigned them to the corresponding group of intervention following the randomization list.

The patients were blind to their type of intervention. Blinding of the operator who performed the procedure (MR) was not possible.

The null hypothesis of the study was that there would be no significant difference in NRS scores over time between the two treatments.

### 2.7. Statistical Analysis

Data were analyzed with R Software (R version 3.1.3, R Development Core Team, R Foundation for Statistical Computing, Wien, Austria). Descriptive statistics (mean, standard deviation, minimum, median, and maximum) were calculated for each group. Data normality of the distributions was calculated with the Kolmogorov–Smirnov test. Subsequently, the ANOVA test for repeated measures was applied, followed by the post hoc Tukey test in case the results were significant.

Significance was set at *p* < 0.05 for all the tests performed.

## 3. Results

**Figure 3:**
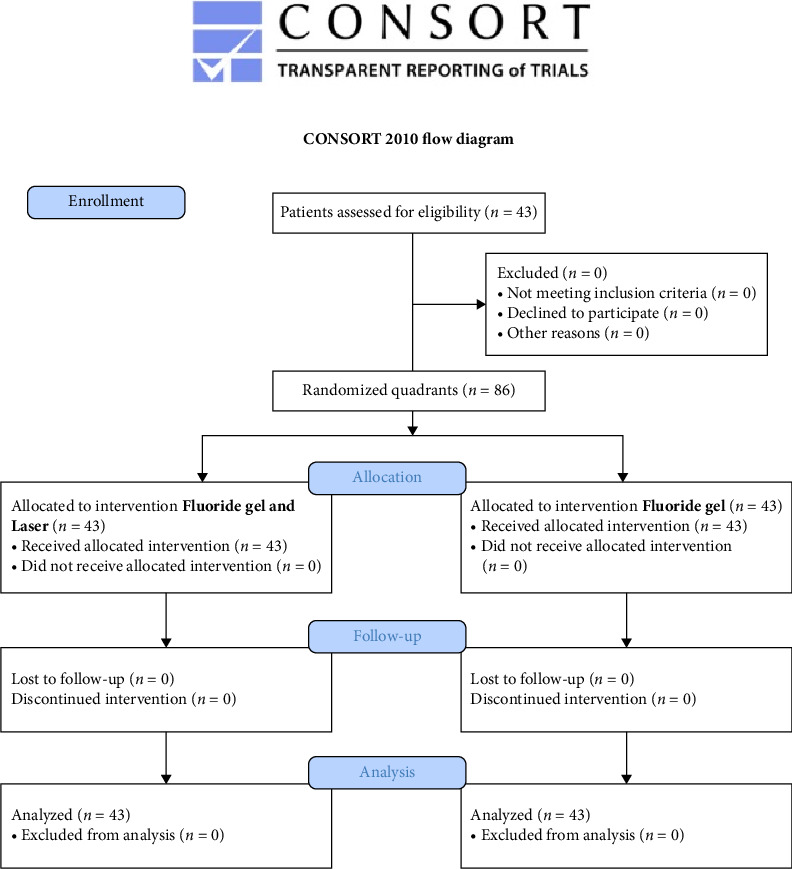
CONSORT flow chart of the study showing enrollment and allocation procedures.

Participants were enrolled until the total number of 43 patients (86 quadrants) was reached. Patients all agreed to participate in the study and received the allocated interventions. No patient was excluded from the analysis. The flow chart of the study is shown in Figure 3.

Table 2 shows the characteristics of the patients included in the study.

**Figure 4:**
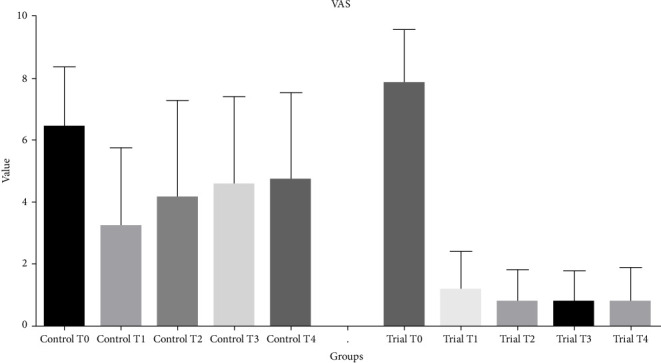
Histogram showing mean and standard deviations of the NRS values in the two groups over time.

ANOVA showed the presence of significant differences among groups (*p* < 0.0001). Post hoc test showed that NRS values significantly decreased from T0 to T1 for both the groups (*p* < 0.0001). In the control group, a gradual increase in the NRS scores was registered over time, and significantly higher scores were registered at T4 if compared with T0 (*p* < 0.0001). Concerning trial group, no significant difference was reported when comparing T1, T2, T3, and T4 values (*p* > 0.2344). As regards intergroup comparisons, no differences were reported between trial and control groups at T0 (*p*=0.27). Trial group showed significantly lower values at T1, T2, T3, and T4 (*p* < 0.0005) as shown in Table 3 and Figure 4.

## 4. Discussion

DH is a poorly managed condition in dentistry, largely due to the difficulty of making an accurate diagnosis due to its multifactorial pathogenesis, but this pathology has a significant impact on the social life of those affected [25]. The increase in this phenomenon, also due to the increase in the life expectancy of the world's population and the greater knowledge and expertise in oral hygiene at home, is leading to an irreversible increase in the demand from patients for a practical and permanent solution to this condition [26].

The null hypothesis of the present trial has been rejected. In fact, combined use of laser and fluoride was reported to be more effective than application of fluoride alone.

Fluorinated substances have always been considered the main therapies for the resolution of hypersensitivity tooth [27–29]. Although these substances are not the only ones reported in the literature to reduce sensitivity, in fact, substances without a fluorinated component, such as casein phosphopeptide–amorphous calcium phosphate (CCP–ACP) nanocomplexes, hydroxyapatite, adhesive systems, propolis, and many others, are useful in these cases. In addition, fluorides have been shown to reduce the bond strength of both restorative [30] and orthodontic [12] materials.

At present, there is no single standardized therapy for this pathology. Apart from therapies that irreversibly affect the dental element (such as root canal therapy and prosthodontic treatment), the only therapeutic options in the office are the use of desensitizing substances, combined or not with laser therapies to increase the effect [31–41].

In fact, DH remains a challenging clinical condition due to its multifactorial etiology and the variability of patient responses to treatment. Several therapeutic approaches have been proposed, aiming either to occlude exposed dentinal tubules or to modulate neural transmission [31, 32]. Among physical strategies, laser therapy has gained attention for its ability to seal tubules and induce analgesic effects through nerve desensitization, although outcomes may depend on the device and protocol employed [33, 34]. Topical agents represent the most widely used approach, particularly desensitizing toothpastes containing compounds such as potassium nitrate, arginine, or stannous fluoride, which reduce tubular fluid movement or stabilize nerve activity even if application with toothbrush could imply slight substrate abrasion [35–38]. Remineralizing agents, including bioactive glasses and calcium–phosphate–based formulations, promote apatite deposition within tubules, thereby restoring mineral content and reducing sensitivity. More recently, biomimetic peptides, such as self-assembling peptides, have demonstrated the ability to form hydroxyapatite-like structures, offering a biologically inspired strategy for long-term occlusion. Additional systems, such as varnishes, adhesives, and iontophoresis, have also been explored, broadening the therapeutic armamentarium [35–41]. Despite the variety of available treatments, clinical studies frequently report heterogeneous results, and no single modality has been consistently identified as superior. Current evidence suggests that while all methods show efficacy in reducing DH, the literature does not support one definitive gold standard.

This is a controversial topic in the literature, but a recent Cochrane review [41] found that, although the level of evidence is low, laser is a useful therapy, even when used alone, to reduce the painful stimulus from air and has no adverse effects in patients undergoing treatment. However, according to other studies [42–45], the association between laser and AF leads to a reduction in the movement of the dental tubular fluid and an increase in the dental inorganic component, which favors a greater closure of the dentinal tubules and therefore decreases the concomitant pain sensation for the patient.

In any case, the densensitizing therapies are all effective in reducing pain, but as other RCTs show [46, 47], the combination of laser and AF shows a clear improvement in pain from DH in a statistically significant way compared to the other groups. Furthermore, this reduction in sensitivity seems to be better maintained over time. Previous reports are partially in agreement with this result, even if a direct comparison between the use of AF alone or in combination with the laser is still not fully investigated [47–49].

Although this study undoubtedly has limitations, such as the limited population threshold, this study shows that in the sample evaluated, there was a reduction in pain symptoms on the NRS scale (*p* < 0.0001) both in the group treated only with AF (treatment group) and in the group treated with laser associated with AF (control group). In both groups, the intensity of pain did not seem to change over time, even 3 months after treatment, NRS from T1 to T3 (*p* > 0.2344).

After 6 months, however, while the control group showed no differences, the treatment group showed a statistically significant difference in NRS over the same period, NRS T1 and T4 group A (*p* < 0.0001), which would indicate that laser therapy was more stable over time compared to AF therapy alone.

The most interesting event of this study was the significant difference in the reduction of the NRS value between the samples evaluated; in fact, comparing the samples that did not show a significant difference at T0 (*p*=0.27), there was an important, statistically significant difference between the treatment group and the control group, in terms of immediate and long-term pain reduction, for the group treated with laser T1, T2, T3, and T4 (*p* < 0.0001), so the null hypothesis must be rejected.

The limitation of the present study is due to the relatively small sample size and single-center design, which may restrict the generalizability of the findings. Moreover, the absence of operator blinding and the short follow-up period may have introduced potential biases and do not allow definitive conclusions regarding the long-term efficacy of the proposed treatment.

This study has shown that both therapeutic methods are useful in reducing the painful symptoms of DH and represent effective strategies for this condition. However, there is a clear difference in the reduction of pain sensation in favour of the laser-treated teeth, and this reduction appears to be more durable over time. Future research with different populations or testing different materials and times may improve our knowledge in this area. In addition, it would be interesting in the future to test laser treatment in combination with other recently introduced materials such as biomimetic hydroxyapatite [50–52] and CPP-ACP [13, 53, 54].

## 5. Conclusions

AF gel is effective in reducing hypersensitivity, but combining it with laser therapy appears to amplify its effectiveness, reducing treatment times and increasing its effectiveness. For these reasons, their synergy should be recommended for the treatment of these disorders.

## Figures and Tables

**Table 1:** Tooth and their percentages in the study for each type of tooth.

Tooth	Control	Trial	Total
Incisors	13 (15.12%)	16 (18.6%)	29 (33.72%)
Canines	2 (2.33%)	3 (3.49%)	5 (5.82%)
Premolars	28 (32.55%)	24 (27.91%)	52 (60.46%)
Total	43 (50.0%)	43 (50.0%)	86 (100.0%)

**Table 2:** Demographic characteristics of the patients included in the study.

Gender	*n*	%	Mean age	Standard deviation
Male	15	34.88	35.8	13.42
Female	28	65.12	38.04	15.92
Overall	43	100.00	37.25	14.97

**Table 3:** Descriptive and inferential statistics of NRS values in the two groups (control: AF gel; trial: AF gel and laser) over time (T0: before treatment; T1: 1 week after treatment; T2: 1 month after treatment; T3: 3 months after treatment; and T4: 6 months after treatment).

Group	Mean	SD	Min	Mdn	Max	Significance*⁣*^*∗*^
Control T0	6.41	1.97	2	7	10	A
Control T1	3.19	2.57	0	4	8	B
Control T2	4.12	3.17	0	5	10	B
Control T3	4.51	2.91	0	5	9	B
Control T4	4.70	2.83	0	5	9	B
.						
Trial T0	7.84	1.74	3	8	10	A
Trial T1	1.13	1.26	0	1	4.5	C
Trial T2	0.76	1.05	0	0	4.5	C
Trial T3	0.73	1.05	0	0	4.5	C
Trial T4	0.73	1.15	0	0	4.5	C

*⁣* ^*∗*^Post hoc results: means coded with the same letters are not significantly different (*p* > 0.05). Means coded with different letters are significantly different (*p* < 0.05).

## Data Availability

The data that support the findings of this study are available from the corresponding author upon reasonable request.
